# Circadian rhythm disruption and immunometabolic dysregulation in cancer: rethinking chronotherapy in precision oncology

**DOI:** 10.1097/MS9.0000000000004515

**Published:** 2025-12-09

**Authors:** Syeda Tayyaba, Umair Ali, Muhammad Ijaz Khan, Iman Osman Abufatima

**Affiliations:** a Department of Medicine, Azad Jammu and Kashmir Medical College, Muzaffrabad, Pakistan; b Department of Pharmacy, University of Swabi, Swabi, Pakistan; c Faculty of Medicine, University of Medical Sciences and Technology, Khartoum, Sudan

**Keywords:** cancer, chronotherapy, circadian rhythm, immunometabolism, tumor immunity

## Abstract

Circadian rhythm disruption has emerged as a significant yet underexplored factor contributing to cancer initiation, progression, and therapeutic resistance. The circadian clock regulates approximately 40% of the human genome, orchestrating immune function, metabolism, and cellular repair. Aberrant expression of clock genes alters tumor metabolism and immune surveillance, promoting tumor growth and immune evasion. Understanding the role of circadian misalignment in cancer biology could pave the way for optimizing drug delivery, improving immune checkpoint therapy outcomes, and advancing personalized chronotherapeutic interventions.

*Dear editor,*

The circadian rhythm, governed by transcriptional-translational feedback loops involving *CLOCK, BMAL1, PER*, and *CRY* genes, coordinates vital physiological processes such as metabolism, immune regulation, and cell division[1]. Disruption of these rhythms due to environmental, behavioral, or genetic factors is increasingly recognized as a hallmark of carcinogenesis[2]. Recent studies have demonstrated that loss of circadian synchronization enhances tumor proliferation, metabolic reprogramming, and resistance to therapy[3]. Evidence from transcriptomic and metabolomic analyses indicates that circadian dysregulation reprograms the tumor immune microenvironment. Decreased *BMAL1* expression suppresses T-cell cytotoxicity, while altered *PER2* signaling leads to immune exhaustion. A 2025 study by Zhang *et al* in *Nature Medicine* reported that circadian misalignment impaired interferon-γ secretion and CD8⁺ T-cell function in melanoma-bearing mice, significantly accelerating tumor growth[4]. The restoration of circadian rhythm through time-restricted feeding and light–dark synchronization reversed these effects, underscoring the link between temporal homeostasis and immune fitness. Chronotherapy, the strategic alignment of drug administration with biological rhythms, has gained renewed attention. Several anticancer drugs including oxaliplatin, doxorubicin, and cisplatin demonstrate time-dependent efficacy and toxicity patterns[5]. Optimizing drug delivery schedules in accordance with patient chronotype may enhance therapeutic index and reduce systemic toxicity. A recent multicenter clinical trial integrating chronotherapy with PD-1 inhibitors in advanced malignancies observed improved overall survival and reduced immune-related adverse events[6]. At the molecular level, clock genes modulate tumor metabolism via regulation of NAD⁺ biosynthesis, mitochondrial respiration, and glycolytic flux. Circadian disruption enhances the Warburg effect and promotes metabolic immunosuppression through increased lactate accumulation[7]. These findings highlight that the circadian clock is not merely a passive temporal regulator but an active participant in cancer immunometabolism. Despite these insights, implementation in clinical oncology remains limited. Heterogeneity in patient chronotypes, lifestyle patterns, and genetic polymorphisms complicates standardization of circadian-based interventions. Developing reliable biomarkers such as melatonin profiles, core body temperature rhythms, or transcriptomic clock signatures could enable individualized therapy timing. In conclusion, circadian rhythm disruption represents an untapped therapeutic axis in precision oncology. Integrating chronotherapy protocols, circadian biomarker profiling, and immune-metabolic readouts into cancer trials could revolutionize therapeutic optimization. The intersection of temporal biology and oncology warrants further exploration as a cornerstone of future cancer treatment paradigms.

This manuscript complies with the TITAN Guidelines, 2025, declaring no use of artificial intelligence[8].

## Data Availability

All data supporting the findings of this study are available within the article. The study is based on previously published literature; no new data were generated or analyzed.
